# A descriptive analysis of the role of a WhatsApp clinical discussion group as a forum for continuing medical education in the management of complicated HIV and TB clinical cases in a group of doctors in the Eastern Cape, South Africa

**DOI:** 10.4102/sajhivmed.v20i1.982

**Published:** 2019-08-01

**Authors:** Joana Woods, Michelle Moorhouse, Lucia Knight

**Affiliations:** 1 Wits Reproductive Health and HIV Institute (WRHI), Johannesburg, South Africa; 2 School of Public Health, University of the Western Cape, Cape Town, South Africa

**Keywords:** Continuing medical education, HIV/TB, Eastern Cape, WhatsApp, Clinician

## Abstract

**Background:**

As South Africa’s (SA) HIV programme increases in size, HIV/TB cases occur that are often beyond the clinical scope of primary healthcare clinicians. In SA’s Eastern Cape (EC) province, health facilities are geographically widespread, with a discrepancy in specialist availability outside of academic institutions. The aim of this study is to describe WhatsApp and its use as an alternative learning tool to improve clinicians’ access to specialised management of complicated HIV/TB cases.

**Objectives:**

To analyse clinicians’ use of the WhatsApp chat group as a learning tool; to assess clinicians’ confidence in managing complicated HIV and TB patients after participating in the WhatsApp case discussion group; to describe the perceived usefulness of the chat group as a learning tool; to understand clinicians’ knowledge and use of informed consent when sharing patient case details on a public platform such as WhatsApp.

**Method:**

An observational, cross-sectional study was conducted among a group of clinicians from the EC that formed part of a WhatsApp HIV/TB clinical discussion group. Data were collected using a structured anonymous Internet questionnaire and analysed with Epi Info, using descriptive and analytic statistics.

**Results:**

The analysis found the majority of participants had gained new clinical confidence from group participation. This was associated with the increased group engagement in group follow-up (odds ratio [OR] 48.13 [95% confidence interval [CI] 4.99–464.49]); in posting questions (OR 3.81 [95% CI 1.02–18.48]); in reports of ‘new’ clinical insights (OR 23.75 [95% CI 3.95–142.88]); in referencing old case material (OR 21.42 [95% CI 4.39–104.84]) and in the use of peer guidance to manage cases (OR 48.13 [95% CI 4.99–464.49]). However, there was a discrepancy in participants’ knowledge and actual use of informed consent when posting patient details on social media.

**Conclusions:**

Our study findings support the use of WhatsApp in a medical setting as an effective means of communication, long distance learning and support between peers and specialists.

## Background

Many nations face problems of inequitable access to healthcare services and the shortage of suitably qualified healthcare professionals. An insufficient number of medical graduates; a scarcity of postgraduate education; the migration of healthcare professionals and a critical shortage of teaching faculty demonstrate a need for alternative approaches to improving the retention of the healthcare workforce.^1^ A possible contributing solution to this problem is found in continuing medical education (CME).

Countries must retain health professionals by providing them with opportunities for career development, CME, motivation and support.^2^ The evidence shows that career development and CME strongly motivate health professionals to stay in their own countries and to practise in remote areas.^3^ However, many health professionals struggle to access CME because of professional isolation, lack of locum relief and heavy workload, and this is seen particularly in rural areas.^1^ Much CME traditionally happens through conferences, seminars and other face-to-face meetings. These are often difficult to attend which limits training to attendees only.^1^ This is particularly applicable to clinicians working in the Eastern Cape (EC), a predominantly rural province, where the clinicians enrolled in this study are working.

In South Africa, 46% of the population live in rural areas, but only 19% of the nursing workforce and 12% of physicians practise in those areas.^2^ The EC has a population of 7 million and an HIV prevalence of 12.1%.^4^ Approximately 4.1 million of the population live in rural communities.^5^ In this setting, district hospitals and public health clinics are often geographically widespread, with only three academic or tertiary centres servicing these facilities.^6^ In addition, the province has only four infectious disease (ID) specialists to provide expert care to its seriously ill HIV and TB patients.

Per population size, South Africa (SA) has the largest HIV epidemic in the world. The overall HIV prevalence rate is approximately 12.6%. Similarly, the country’s TB burden is large. In 2016, SA recorded 438 000 new TB infections.^7^ TB was the leading cause of death in the country. HIV treatment and care is often complicated by the emergence of drug resistance, drug–drug interactions and the advanced immune suppression of newly diagnosed patients.

The use of smartphone technology and MIM platforms in clinical practice is a research topic that is gaining support. Since January 2017, there are 1.2 billion active WhatsApp users worldwide.^8^ This service offers users the following features: the transmission of text messages, images and videos to contacts and a chat group feature that allows 256 users to share content simultaneously.^9^

However, its use in the public health sector has been poorly researched with only a small number of studies published.^10^ The literature that is available shows that the use of this technology offers an efficient, unobtrusive and portable mode of communication for medical staff.^11^ Not only that, but also medical images that are captured using smartphone devices promote the delivery of medical care in a timely and resource-friendly manner.^12^ Kankane et al., in a study of neurosurgical communication, found that WhatsApp enabled cost-effective and quick decision-making, namely 4.06 min from image to registrar report.^13^ This led to earlier diagnosis and more prompt treatment. Nikolic et al. suggest that this technology has the potential to improve patient education and management, and perhaps, to impact significantly on health provision as a whole.^11^

There are obvious concerns, however, about the transmission of confidential patient information over a social media platform. According to international guidelines, patient confidentiality and guarding their personal health data are a legal requirement under different laws, such as the *Health Information Portability and Accountability Act* (HIPAA) in the United States, or the Data Protection Directive in the European Union.^14^ There are currently no Health Professionals Council of South Africa (HPCSA) guidelines that address the issue of clinicians specifically posting on social media. However, their guidelines address the issue of patient confidentiality, as well as ethical concerns using telemedicine (which have been extrapolated below to the use of social media). Clinicians who wish to publish details about specific medical cases or clinical experiences online, which identify or run the risk of identifying a patient, should ensure they follow the guidelines relating to patient consent and disclosure set out by the HPCSA.^15^ These state that a patient’s express consent must be obtained before publishing case reports, photographs or other images in media that the public can access. WhatsApp has improved its end-to-end encryption policies and does not store chat data in a virtual cloud (like Facebook), but this form of protection has not been conclusively tested in clinical environments. Patient confidentiality is therefore still at risk. The increased use of medical social media, data and information can be very useful, but any abuse of data needs to be prevented.^14^

A WhatsApp messenger chat group was created in 2016 for doctors who had attended an advanced HIV management course, and were working in district hospitals in the EC. The group included medical specialists and members of the district clinical support team. Clinicians posted complicated cases. The discussion that followed referenced national and international HIV guidelines and evidence-based clinical care. This provided cumulative medical expertise that assisted the clinician in the management of the case.

It is important to know if this intervention is of benefit to doctors, particularly those without onsite specialist support in the South African healthcare context. It is also important to know if clinicians are aware of local occupational governing authority rules relating to patient confidentiality breaches when posting on social media. This would raise awareness of these important ethical and legal obligations in the medical fraternity. The data obtained from this research could be used to motivate for the use of alternative platforms of learning and clinician support across different medical specialist modalities besides ID care. This intervention could then support the World Health Organization’s (WHO) recommendation that countries can aid in the retention of health professionals by providing them with opportunities for career development, CME, motivation and support.^16^

## Aim and objectives

### Aim

The aim of this study is to describe the use of a WhatsApp clinical discussion group as an alternative learning tool to improve clinician access to specialised clinical management of complicated HIV/TB cases, as part of CME, and their knowledge of informed consent use when posting patient cases on social media.

### Objectives

The specific objectives are:

- to analyse clinicians’ use of the WhatsApp chat group as a learning tool
- to assess clinicians’ confidence in managing complicated HIV/TB patients after participating in the WhatsApp case discussion group
- to describe the perceived usefulness of the chat group as a learning tool
- to understand clinicians’ knowledge and use of informed consent when sharing patient case details on a public platform such as WhatsApp.

## Research methods and design

### Study design

An observational, descriptive cross-sectional design was used, with an anonymous Internet questionnaire, distributed to the clinicians who formed part of the WhatsApp group, as the data source. A quantitative approach was chosen for the study as the responses from the questionnaire were graded and therefore easily quantifiable.

### Study population, setting and sampling

The study population that was used in this study were 166 doctors from the EC province that accepted the organiser’s invitation to be part of the WhatsApp clinical discussion group from January 2016 to July 2017. The inclusion criteria for the study included doctors from the EC Department of Health, as well as clinicians from collaborating non-governmental organisations (NGOs). All the 166 doctors in the group were included to minimise any non-response, and to improve representation of the clinicians in the group.^17^

### Data collection tools and collection

Data were collected using a structured, anonymous Internet questionnaire. This comprised 17 statements or questions, each with a corresponding answer or choice of answers. The main themes for the questionnaire centred on access to the WhatsApp or Internet; usage of the group; aid in improving clinical confidence; usefulness as a learning tool and the confidentiality of cases posted (doctors’ perceptions).

To reduce information bias, the investigator used a standardised tool, and each doctor received the same questionnaire. The questionnaire had been reviewed by a group of three colleagues to ensure clarity and the exclusion of external bias. Once ethical approval was received and before distribution to the participants, the questionnaire in its electronic format was piloted with the same colleagues who are a part of the WhatsApp group to further improve question clarity and ease of participation. The questionnaire was self-administered, so no measurement bias was introduced by a third party.^18^

There was a threat to validity in terms of sampling bias when administrating the questionnaire, with the potential of non-responders possibly skewing the results.^17^ Forty-five per cent (74/166) of the participants did, in fact, not submit responses. The investigator attempted to minimise this threat as much as possible by regular email and WhatsApp reminders.^17^ The questionnaire was kept as short as possible and attempts were made to simplify access to it with an easy to use Internet link being sent to the participants – all this to minimise non-responses.^17^

A link to the questionnaire in the Google form was initially sent to each clinician in the WhatsApp discussion chat group via WhatsApp. When the clinicians clicked on the link, they were taken to the electronic Google form. Google saved each completely filled questionnaire in the investigator’s Google drive. This form was completed by the respondent by a click on the most appropriate response. There were no open-ended or continuing questions, making the questionnaire simple and fairly quick to answer; the investigator estimated around 5 to 10 min per form. Participants were able to answer the questions within their own time frame, enabling them to have privacy or choice of space.

All the completed forms were available to view on the drive, which was password protected, and could be downloaded when needed for analysis. The clinicians were also emailed the link as well. Emailing helped to collect data from the clinicians that may have at any stage left the group during the period under investigation.

### Data management and analysis

The individual responses saved on Google drive were collected and transferred to an Excel spreadsheet, where data cleaning occurred. Any incompletely answered questionnaire was removed as a data source. Text responses were also allocated a numerical key for easier analysis. The data were then imported into Epi info statistical programme for analysis and were initially explored using basic frequencies for the categorical data.

Summary statistics were presented to give a general description of the above responses using analysis tables and graphs. These categorical variables were summarised as the number and percentage of responses in each category or exposure variable. Further analysis was done by looking at other possible associations between clinical confidence to group engagement and clinical confidence because of perceived usefulness of the group as a learning tool. In the confidence variable, like–like response options were recorded for ease of analysis. Other associations included the recommendation of the group based on the perceived usefulness of the group as a learning tool. For all the above associations, frequency distributions and cross-tabulations of the above-mentioned variables were generated. Bivariate analysis was done to determine significant associations between the differing variables using *p*-values, odds ratios (ORs) and 95% CIs. The assessment of any significant differences was conducted using a Mid-P Exact test. This was computed to confirm statistical significance. Type I error rate (alpha) for statistical tests was set at 0.05 and 95% CI, and were provided when appropriate.

## Ethical consideration

Ethics approval was received from: HREC (Human Research Ethics Committee) from the University of Witwatersrand; BMREC (Biomedical Research Ethics Committee) from the University of the Western Cape. A participant information form (combined with participant consent), along with the link to this internet questionnaire was electronically available and posted on the WhatsApp group, as well as emailed to all participants who had at any stage belonged to the group within the reporting period. As the questionnaire was anonymous, no participant name was requested. There was no anticipated harm in the study, but there may have been some discomfort to the doctors in completing the online questionnaire. There was also the risk of identifying the locality of where the doctors worked (i.e. EC) but no risk of identifying individual doctors or patients/cases.

## Results

### Sample description

Out of the 166 belonging to the WhatsApp chat group, a total of 92 participants submitted Internet questionnaires. One form was submitted with no answers and was therefore excluded from the analysis.

### Analysing clinicians’ use of the WhatsApp group

To analyse the usage of the WhatsApp group (objective one of the study), the questionnaire included questions that assessed the participant’s Internet accessibility and their engagement in the group. Satisfaction at the relevance of responses received (by content and timing) was also assessed. Lastly, participants were asked what types of cases they posted.

Only 1% of participants did not have access to a form of Internet connectivity. Twenty-nine per cent of the remainder had only occasional access. Internet connectivity and access was important to permit the receipt and posting of questions on the app. Seventy-one per cent of participants had access all the time. The majority (73%) looked on the app every time a case was posted, with only 2% ignoring the group completely.

To further assess engagement in the group, participants were also asked how many times they posted cases in the group, and if they posted any responses to a case that had been posted by a colleague. Half of the participants reported to have never posted cases; 47% had posted at least 1–5 times and 3% had posted 6–10 times. In terms of posting any medical advice or responses to another colleague’s case, 52% posted occasionally, 4% all the time.

To determine the satisfaction of case responses received, participants were asked if the responses to the cases posted came timeously. The participants who had posted cases felt positively about the timely case response. The majority of participants who posted cases also stated that they were satisfied with the content of the case response received.

**FIGURE 1:**
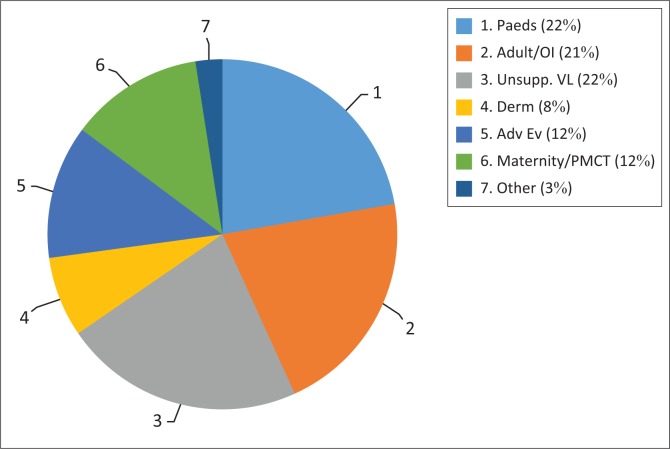
Types of cases discussed (*n* = 81).

Those participants who posted cases were asked what type of cases they presented (Figure 1). There was a very similar distribution in reporting paediatric (Paeds), adult cases (including opportunistic infections [OI]) and cases of unsuppressed viral loads (Unsupp. VL) – making up the bulk of cases at 65% collectively. Other cases discussed included dermatological conditions (Derm), adverse events (Adv Ev), maternal cases and prevention of mother-to-child transmission (PMCT).

Lastly, in terms of participants’ perceptions of having obtained greater clinical confidence in managing complicated HIV/TB cases (study objective two), the majority (86%) agreed that it did increase their clinical confidence.

### Perceived usefulness of the chat group as a learning tool

The questionnaire also assessed the participants’ perceived usefulness of the group as a learning tool in managing complicated cases after taking part in the group (study objective three), and whether they would recommend this case discussion platform to other colleagues.

When participants were asked if they used the clinical guidance posted on the WhatsApp doctors group in their own patient management, 52% responded that they used the clinical guidance all the time, 44% used the guidance occasionally. Only 4% felt that the guidance given on the group was not relevant to their current patient case management. About a third of the participants reported that they actually referred back to old cases discussed all the time when a complicated clinical case presented at their clinic. Out of the remainder, 64% used the previous discussions occasionally, and 8% felt that felt that the guidance given on the group was not relevant to their current patient case management.

Again, the majority of the participants strongly agreed that the WhatsApp group was useful in helping them gain new clinical insights on HIV/TB, that the information discussed in the group chat was according to national guidelines and international best practice principles and they would recommend a similar case discussion platform to other colleagues (Table 1)

**TABLE 1:** Usefulness of the group as a learning tool (*n* = 91).

Variable	Strongly agreed		Agreed		Neutral		Disagreed		Strongly disagreed	
	*n*	%	*n*	%	*n*	%	*n*	%	*n*	%
New clinical insights	51	56	32	35	7	8	1	1	-	0
Practical application of pre-existing knowledge	49	54	34	37	8	9	-	0	-	0
Guidance according to national or international guidelines	57	63	31	34	4	4	-	0	-	0
Recommend to colleagues	58	64	23	25	8	9	-	0	2	2

### Clinicians’ knowledge and use of informed consent in the group

The last objective was understanding the clinicians’ knowledge of informed consent when sharing patient information on social media. From the responses, 89% of the participants reported that they were aware that according to HPCSA regulations, they needed to obtain documented patient consent when posting a patient-related image on social media. However of those that reported posting questions, only half obtained consent (52%) versus 48% not obtaining consent, when posting patients’ photographs or other medical images on the group (even if patient identity was not revealed). When asked if they obtained documented patient consent when posting patients’ laboratory results on the group (even if patient identity was not revealed), around two-thirds (68%) of participants who had posted said they had in fact not obtained consent, less so than when posting other medical images.

### Bivariate analysis

Using a bivariate analysis, with cross-tabulation in Epi Info, any statistically significant associations were looked for in those clinicians who reported feeling more confident in managing their patients after group participation and whether they would recommend the group as a learning platform to other colleagues.

Table 2 looks at any association between group engagement as an exposure variable, and increased clinician confidence as an outcome. In doctors who followed the group regularly, there was a clinically significant increase in OR (8.44, 95% CI 2.33–35.23), participants being 8.44 times more likely to have increased confidence in managing their patients. Those who posted questions also had an increase in OR, 3.8 times more likely to have an increase in their clinical confidence (95% CI 1.02–18.48).

**TABLE 2:** Increased clinician confidence in managing patients and levels of group engagement.

Variable	Greater clinical confidence (%)	OR	95% Confidence interval	*p*
Followed group	93	8.44	2.33–35.23	< 0.05
Posted questions	93	3.8	1.02–18.48	0.02
Posted responses	92	3.36	0.96–13.55	0.03

OR, odds ratios

Other associations were found in increased clinician confidence in managing patients as an outcome, cross-tabulated with participant perceptions of the usefulness of the group as a learning tool (Table 3). Of statistical significance, participants who used the chat group guidance to manage their patients were 48.13 times more likely to be confident afterwards (OR 48.13, 95% CI 4.99–464.49); those who referred to old chat cases were 21.42 times more confident (OR 21.42, 95% CI 4.39–104.84); there was also an increase in confidence in participants who reported that they had gained new clinical insights while participating in the group (OR 23.75, 95% CI 3.95–142.88).

**TABLE 3:** Increased clinician confidence in managing patients based on their perceived usefulness of the group as a learning tool (*n* = 91).

Variable	Greater clinical confidence (%)	OR	95% Confidence interval	*p*
Used guidance to manage patients	91	48.13	4.99–464.49	< 0.05
Referred to old chat cases	91	21.42	4.39–104.84	< 0.05
New clinical insights	90	23.75	3.95–142.88	< 0.05
Practical application of pre-existing knowledge	94	Undefined	Undefined	< 0.05
Guidance according to national or international guidelines	89	Undefined	Undefined	< 0.05

OR, odds ratios.

When looking at recommending the group to colleagues as an outcome, participants who report gaining new clinical insights were 17.33 times more likely to recommend the group (95% CI 3.13–96.01). Those who reported that the group helped them to practically apply pre-existing knowledge and felt that the guidance given was according to national or international guidelines were also, respectively, 12.82 (95% CI 2.55–64.56) and 20 (95% CI 1.63–245.63) times more likely to recommend the group to other colleagues as a case discussion platform (Table 4).

**TABLE 4:** Clinician recommendation of the WhatsApp group based on their perceived usefulness of it as a learning tool (*n* = 91).

Variable	Recommend group (%)	OR	95% Confidence interval	*p*
Used guidance to manage patients	89	1.69	0.17–16.1	0.32
Referred to old chat cases	90	2.64	0.46–14.95	0.16
New clinical insights	93	17.33	3.13–96.01	< 0.05
Practical application of pre-existing knowledge	93	12.83	2.55–64.56	< 0.05
Guidance according to National or international guidelines	91	20	1.63–245.63	< 0.05

OR, odds ratios

In terms of group engagement and recommending the group to others, those who followed the group regularly were 4.79 times more likely to recommend it (95% CI 1.19–21.10). There was no difference in those who posted questions and responses (Table 5).

**TABLE 5:** Clinician recommendation of the WhatsApp group based on their level of engagement in the group (*n* = 91)

Variable	Recommend group (%)	OR	95% Confidence interval	*p*
Posted responses	88	0.83	0.19–3.28	0.4
Posted questions	88	0.97	0.24–3.89	0.46
Followed group	93	4.79	1.19–21.10	0.01

OR, odds ratios

Lastly, we looked at whether Internet access impacted clinicians’ reported ability to follow the group, but there was no clinically significant association found.

## Discussion

### Group engagement and its usefulness as a learning tool

The responses from participants in this study were favourable in the reported use of the WhatsApp group and its application as a learning tool. The majority of the participants firstly cited regular Internet connectivity, which facilitated the uninterrupted use of the application and communication in real time.^19^ They also reported using the group discussions as a guide to further manage other patients, referred back to old chat discussions and were satisfied at the timeous response to cases (including the peer responses themselves). Group engagement, or participation, measured by following of the group and posting of cases or responses, was regular. The results further demonstrated that there was a statistically significant association between engagement in the group and increased clinical confidence – those who followed the group regularly were 8.44 times more likely to report an increase in clinical confidence and 3.8 times more confident if they posted a case. This correlates with findings by Raiman et al., who discussed in their article that the use of mobile applications has been shown to increase student participation and therefore foster improved learning.^20^ Similarly, Rambe et al. also reported that WhatsApp facilitated, in learners, the ability to more confidently engage with peers and educators.^19^

This form of engagement and success can be described through the theory of cooperative learning. In cooperative learning, students who maximally engage in a group are able to extend their current knowledge base, as they are in control of the discussion construct.^21^ In our study’s context, participants posted a case they are most interested in and there develops a close relationship between theory, research and a practical working through the case; this underpins long-term retention of knowledge and maximises student learning.^21^

Furthermore, the participants also reported that the group gave them new clinical insights; helped them to practically apply pre-existing knowledge and felt that the guidance was aligned with international or national guidelines. In a systemic review of medical literature, Kamel Boulos et al. found collective evidence that WhatsApp has been successfully used in health and medical education and learning.^9^ They also concluded that apps can help to create virtual communities of enquiry and practice, and bridge distances of busy distributed healthcare settings. Our research adds to the literature by further clarifying that the knowledge gained (whether from peers or specialists) in belonging to such a group aids in the application of new clinical insights and previous medical knowledge, as well as contributing to clinical confidence by facilitating distance learning.

Lastly, our study found that participants were more likely to recommend the group to other colleagues if they had followed the group regularly (OR 4.79), and in those who reported the group as a useful learning tool. The investigators therefore surmised that the WhatsApp group seems to have promoted good group engagement which, in turn, facilitated learning, decreasing professional isolation and produced a recommendation of a similar platform to other colleagues. Such a mobile learning platform is therefore an important adjunct to current CME practices. E-learning (of which WhatsApp forms a part) can result in greater educational opportunities for participants, while at the same time enhancing student effectiveness and efficiency, as is the reported outcome in our study.^22^

### Clinical confidence in managing complicated HIV and TB cases

The majority of the participants in the study agreed that they had gained greater clinical confidence in managing their patients after participating in the group. The findings showed that there was also an improvement in clinical confidence among those participants who perceived the group as a useful learning tool (it has been previously mentioned how engagement in the group had a similar effect). Participants who used the chat group guidance to manage their patients were 48 times more likely to feel clinically confident. There was an increase in clinical confidence in those who referred to old chat cases (OR 21.42) and those who gained new clinical insights while participating in the group (OR 23.75). Raiman et al. reported similar findings in their study – a WhatsApp group provided a unique environment to be able to quickly access learning resources while participating in a discussion facilitated learning. Their participants also cited how useful it was to look back at old recorded learning discussions.^20^

Improved clinical confidence among our participants could be because of two main aspects: accessibility and case-based learning. Doctors could easily access the application, could easily access old cases in the application and could easily access new knowledge by asking for guidance on the application. Wani et al. found that doctors started management of patients quicker after using WhatsApp clinician advice because of faster access to that advice, and that they found that management to be more effective.^23^ It is often laborious trying to find best evidence-based management in medical literature, especially in a time-constrained clinical setting. Also, the application of that knowledge is sometimes broad, with medical theory not always correlating clearly to what is found in clinical practice. By providing input on a specific case (in a specific South African clinical setting) and supporting the clinician in managing the case in real time, a clinician’s confidence can be further bolstered. The WhatsApp group provides a form of case-based learning, which has been shown to tie theory to practice and promote deeper learning.^24^ Studies that use interactive techniques for CME, such as case discussions, produce a favourable change in professional practices and outcomes.^25^ This correlates to a similar reported outcome in our study.

### Clinicians’ knowledge and use of informed consent

Although WhatsApp is relatively safe in terms of hacking and leaking of confidential content because of its end-to-end encryption of data, there is still much concern about its use in medical literature and the impact on patient confidentiality.^26^ The majority of our study participants reported being aware that they needed to obtain documented patient consent when posting a patient-related image (photographs, case report, laboratory report) on social media. However, less than half of participants actually obtained consent. There seems to be a discrepancy in what the clinicians reported to know, and what they did to maintain patient confidentiality in this study.

Several authors share similar concerns that the use of patient data needs to be regulated when using social media, and that there needs to be a review of the roles and responsibilities of medical professionals when using such platforms.^14^ Mars and Escott found few reports of patient consent being regularly sought when sending patient information over WhatsApp.^26^ They concluded that doctors need to be told what steps to take to maintain confidentiality. In our study’s WhatsApp group, group rules were posted advising clinicians to remove any patient identifiers from any medical images when posting. This helped in some ways towards preserving patient confidentiality, but further education needs to be iterated to our study group regarding obtaining actual documented consent from the patients themselves.

### Generalisability

The results of the study show that WhatsApp is perceived as an effective means of learning and clinical support in this study group. This mobile application can then be applied to other clinical disciplines (not only IDs), from other health settings (private, district, provincial level), as a learning intervention. The target population groups that could potentially use this intervention include, for example, doctors in other clinical disciplines who need expert advice or access regarding patient management, allied health professionals (such as nurses, physiotherapists, occupational therapists) who need clinical supervision and advice from senior consultants regarding patients they are managing. As this study looks at the use of the WhatsApp group in a clinical setting for patient management and further medical learning, it would be difficult to comment if its use would be applicable outside of the medical field.

The second outcome of the study was to assess if patient confidentiality breaches had occurred with the posting of cases, and if doctors were aware of the legal obligations they are under when posting patient case details on a social media platform. The findings of this study could definitely be generalised to any health profession. It would aid in raising awareness of the pitfalls of posting cases on social media, and in doing so, protect health professionals from any future litigation as well as protecting their patient’s confidentiality.

### Limitations

There are several limitations to this study. Although many attempts were made to get responses from the group, only 55% (*n* = 91) of participants submitted responses; the selection was therefore not random, and could introduce some bias. The small sample size and simple survey framework could affect the overall results, with resultant wide CIs. Some further bias could have been introduced by the online format of the questionnaire (with possible technical inability to fill it in correctly). A possible bias might have also occurred if any of the collaborating NGO clinicians filled in the questionnaire, although only four were active in the group at the time. We did not collect any demographic data from the respondents. The retrospective nature of the study could also affect the participants’ responses, as recall of their experiences of the chat group over a period of 1 year could vary from their original experiences. The investigators could also not determine from the study if the responses received to posted questions were from peers or specialists. A more accurate observation of the WhatsApp group would have been obtained through direct analysis of the chat contents, but this was not approved by local ethical governing bodies without written consent from each doctor (which would be beyond our scope given the study’s retrospective nature).

## Conclusion

The initial aim of this study was to show that participating in a WhatsApp group was a useful adjunct learning tool that could also clinically support doctors in geographically widespread facilities without onsite specialist support. Based on the participant responses from this research, this mobile platform does offer an alternative CME solution that can be easily and successfully implemented in various health fields. By giving healthcare professional opportunities for career development, CME, motivation and support through this novel learning platform, we can perhaps aid in their retention in the public health sector.^16^ Caution needs to be taken to maintain patient confidentiality when posting on social media, but that does not negate WhatsApp’s usefulness in a clinical learning setting.

## Recommendations

The investigators recommend the use of WhatsApp clinical support groups as a long-distance learning platform, based on our findings. To facilitate group success, some further recommendations include a commitment from participants in the group with active participation; a case-based method of discussion (but other learning modalities can be used); cooperative engagement led by students determining the learning construct of the group that will benefit them the most; and a range of different levels of clinical expertise in the group. There needs to be an increased awareness and education among clinicians on the legal implications of posting patient details in social media (not just WhatsApp) without proper informed consent, to protect patient confidentiality. We also suggest that further research should be conducted to obtain a more objective analysis as to whether advice given in these mobile platforms improves clinical management in patients or not. These could include auditing clinical advice given on clinician WhatsApp groups, according to best practice principles in medical literature, or by directly auditing patient outcomes in those having been managed by doctors who participate in similar mobile learning platforms in South Africa. The findings of this study will be posted on the WhatsApp group, which is still ongoing.

## References

[1] Lygidakis H, McLoughlin C, Patel K Achieving universal health coverage: Technology for innovative primary health care education. 2016 10.13140/RG.2.2.35291.57121

[2] Buchan J, Couper I, Viraj T, et al Early implementation of WHO recommendations for the retention of health workers in remote and rural areas. Bull World Health Organ. 2013;91(11):834–840. 10.2471/BLT.13.119008 24347707 PMC3853959

[3] Chen L Striking the right balance: Healthcare workfore retention in remote and rural areas. Bull World Health Organ. 2010;88(5):323 10.2471/BLT.10.078477 20461215 PMC2865673

[4] Stats SA Mid-Year Population Estimates 2017 [homepage on the Internet]. 2017 [cited 2017 Jul 01]. Available from: https://www.ons.gov.uk/peoplepopulationandcommunity/populationandmigration/populationestimates/datasets/populationestimatesanalysistool.

[5] HSRC Safe hygiene practices in Eastern Cape rural communities. 2012.

[6] Gray A, Vawda Y South African health review 2017; 2017 ISSN 978-1-919839-89-9

[7] SANAC Let our actions count: South Africa’s national strategic plan for HIV, TB and STIs 2017–2022. South African Natl AIDS Counc [serial online]. 2017 [cited 2017 Jul 01];1(March):1–132. http://sanac.org.za/wp-content/uploads/2017/05/NSP_FullDocument_FINAL.pdf.

[8] Statistica Number of monthly active WhatsApp users worldwide from April 2013 to January 2017 (in millions) [homepage on the Internet]. Statistica; 2016 Available from: https://www.statista.com/statistics/260819/number-of-monthly-active-whatsapp-users/. [Acessed: 01 July 2017]

[9] Kamel Boulos MN, Giustini DM, Wheeler S Instagram and WhatsApp in health and healthcare: An overview. Futur Internet. 2016;8(3):1–14. 10.3390/fi8030037

[10] Veroni L, Ferrari A, Acerra A Consideration on the use of WhatsApp in physician-patient communication and relationship. Recent Prog Med. 2015;106(7):331–336. 10.1701/1940.21090 26228724

[11] Nikolic A, Anat PD, Wickramasinghe N, Claydon-Platt D The use of communication Apps by medical staff in the Australian health care system: Survey study on prevalence and use. JMIR Med Inform. 2018;6(1):1–7. 10.2196/medinform.9526 PMC5889814 29426813

[12] Kirk M, Hunter-Smith SR, Smith K, Hunter-Smith DJ The role of smartphones in the recording and dissemination of medical images. J Mob Technol Med. 2014;3(2):40–45. 10.7309/jmtm.3.2.7

[13] Kankane VK, Jaiswal GGT Application of WhatsApp: A quick, simple, smart and cost competent method of communication in neurosurgery. Rom Neurosurg. 2016;30(12):306–312. 10.1515/romneu-2016-0049

[14] Denecke K, Bamidis P, Bond C, et al Ethical Issues of Social Media Usage in Healthcare. IMIA Yearb Med Inform. 2015;10(1):137–147. 10.15265/IY-2015-001 26293861 PMC4587037

[15] HPCSA General ethical guidelines for the health care professions, Booklet 14 [homepage on the Internet]. HPCSA; 2008 [cited 2017 Jul 01]. Available from: www.hpcsa.co.za/Uploads/editor/UserFiles/downloads/conduct_ethics/rules/generic_ethical_rules/booklet_10_confidentiality_protecting_and_providing_information.pdf.

[16] WHO Transforming and scaling up health professionals’ education and training: World Health Organization guidelines 2013. Guidelines. 2013:124 10.1613/jair.301 26042324

[17] Levin KA Study design III: Cross-sectional studies. Evid Based Dent. 2006;7(1):24–25. 10.1038/sj.ebd.6400375 16557257

[18] Hernández B, Velasco-Mondragón HE Cross-sectional surveys. Salud Publica Mex. 1987;42(5):447–455. 11125630

[19] Rambe P, Bere A Using mobile instant messaging to leverage learner participation and transform pedagogy at a South African University of Technology. Br J Educ Technol. 2013;44(4):544–561. 10.1111/bjet.12057

[20] Raiman L, Antbring R, Mahmood A WhatsApp messenger as a tool to supplement medical education for medical students on clinical attachment. BMC Med Educ. 2017;17(1):1–9. 10.1186/s12909-017-0855-x 28061777 PMC5219809

[21] Johnson DW, Johnson RT, Smith K The state of cooperative learning in postsecondary and professional settings. Educ Psychol Rev. 2007;19(1):15–29. 10.1007/s10648-006-9038-8

[22] Kinfu Y, Vovides Y, Talib Z, et al The health worker shortage in Africa: Are enough physicians and nurses being trained? Bull World Health Organ. 2009;87(3):225–230. 10.2471/BLT.08.051599 19377719 PMC2654639

[23] Wani S, Rabah S, AlFadil S, Dewanjee N, Najmi Y Efficacy of communication amongst staff members at plastic and reconstructive surgery section using smartphone and mobile WhatsApp. Indian J Plast Surg. 2013;46(3):502 10.4103/0970-0358.121990 24459338 PMC3897093

[24] McLean SF Case-based learning and its application in medical and health-care fields: A review of worldwide literature. J Med Educ Curric Dev. 2016;3:39–49. 10.4137/JMECD.S20377 PMC5736264 29349306

[25] Davis D, O’Brien M, Freemantle N, Wolf F, Mazmanian P, Taylor-Vaisey A Impact of formal continuing medical education: Do conferences, workshop, rounds and other traditional continuing medical education activities change physician behavior or health car outcomes? JAMA. 1999;282(9):867–874. 10478694 10.1001/jama.282.9.867

[26] Mars M, Escott R WhatsApp in clinical practice: A literature review. Stud Health Technol Inform. 2016;231(March):82–90. 10.3233/978-1-61499-712-2-82 27782019

